# Editorial for the Special Issue “Advanced Research in Plant Metabolomics”

**DOI:** 10.3390/cimb45080423

**Published:** 2023-08-14

**Authors:** Chiara Roberta Girelli

**Affiliations:** Department of Biological and Environmental Sciences and Technologies, University of Salento, Provincia le Lecce-Monteroni, 73100 Lecce, Italy; chiara.girelli@unisalento.it

The study of plant metabolome and the role of cellular pathway end products has gained increased attention. Analytical techniques coupled with statistical methods are successfully employed in plant functional genomics for biological and crop physiology applications. Significant research papers and reviews, collected in the Special Issue of CIMB “Advanced research in plant metabolomics”, focused on the plant metabolome, genome and transcriptome characterization after environmental stress, for possible plant crop yield and quality improvement and for phytochemicals characterization aimed at medical purpose.

Among external stresses, Low temperature with Low light [LL] are known to induce photosynthesis inhibition and oxidative stress in pepper plants, affecting crop production. Ding et al. [1] described the role of carotenoid zeaxanthin to mitigate LL stress by reducing ROS accumulation with the enhancement of antioxidant enzymes as a consequence of the related genes up-regulation in pepper plants. The improved tolerance of pepper seedlings to LL stress resulting from zeaxanthin foliar treatment could represent useful basis for further studies on the resistance of peppers to abiotic stress. Among environmental stress, the review by Li et al. [2] focused on the genome-editing technologies application for temperature, drought, and salinity stresses tolerance enhancement of crops. In particular, the application of clustered regularly interspaced short palindromic repeat/Cas9 [CRISPR/Cas9] and transcription activator-like effector nucleases [TALENs] were described to discover the genes involved in response to abiotic stresses resulting in a potential tool for obtaining quality and yield-improved crops. Transcriptomics studies related to plant microRNAs and phytohormones pathway regulation linked to drought stress were reported in the review by Amhad et al. [3]. In particular, findings from available literature suggested as genetic improvement base on microRNAs regulation of plant hormones could be an effective response to water scarcity stress. Metabolic reprogramming and plant hormones content upon insect feeding was then examined in the research paper by Zhao et al. [4]. This metabolomic study focused on the Chinese pine metabolome change after caterpillar feeding and mechanical wounding by employing an Ultra-Performance Liquid Chromatography-tandem Mass Spectrometry [UPLC-MS/MS] platform with extensive targeted metabolomic techniques. The observed variation in the metabolome and phytohormones content induced by feeding stimulation provided useful insights into pathways involved in insect stress-resistance formation in conifers.

Omics technologies was also used in relevant research papers and review aimed to crops quality and yield increase. In particular: the effect of naphthylacetic acid (NAA), commonly used to improve the tuberous root production of *Rehmannia glutinosa*, was analyzed focusing on its regulatory mechanism by Li et al. [5]. Due to its important economic value, since this perennial plant is traditionally used in traditional Chinese medicine, the treatment with foliar spraying of NAA, to increase root yield is commonly applied. By using transcriptome sequencing and Liquid Chromatography-Mass Spectrometry (LC-MS) based metabolomic technique, the authors suggested as the NAA treatment could affect the root quality by changing metabolite content with up/down regulation of quality related genes.

Expression of gene was also investigated by Chen et al. [6]. in a research paper where the effect of phosphorus (P) fertilizer on cadmium (Cd) detoxification mechanism in rice crop was described. Transcriptome and Real-Time Quantitative-PCR analyses showed as P fertilizer application could be able to reduce Cd toxicity by acting either on signaling molecules and Cd transporter genes. Thus, new insights into research of P-Cd interactions regulation mechanism in crops was provided. Rice crops quality was also focused in the work by Xiong et al. [7], where the authors studied the metabolites related to rice quality analyzing three conventional japonica varieties. A non targeted LC-MS/MS-based metabolomic approach was used to define rice quality and taste value metabolites aiming to breed new high-quality rice varieties.

Among plants of commercial values, tree species with important role in ecological environment construction and sustainable forestry production were investigated. Xiao et al. [8] reported a targeted LC–MS/MS based metabolomics study with transcriptomic analysis aimed to define the reason for the loss of apical dominance and resulting multi-branching in pine varieties. The authors performed the determination of endogenous phytohormone content by LC–MS/MS and transcriptome sequencing [RNA-seq] on apical meristems of three pine species. The obtained data identified the plant hormones accumulation as responsible for the loss of shoot apical dominance and the formation of multi-branching, and found as the high expression of specific gene could be considered the cause of dwarfing related gene.

On overview of various metabolomic applications for crop quality was presented by Ncube et al [9] In this review, significant works on the use of analytical technologies in combination with statistical methods aimed to identify possible biomarkers and key metabolic pathway for soybean genetic improvement were described.

Furthermore, identification and characterization of phytochemical compounds was the topic of two research papers collected in the present special issue. The cardio-protective activity of a phenolic compound from the black walnut tree was investigated [10]. In particular, the researchers examined the effect of juglone against isoproterenol-induced myocardial injury in rats suggesting as this metabolite could be used a as a therapeutic agent. An evergreen shrub, widely used for medical applications, *Daphne odora*, was studied by Eom et al. [11], focusing on the depigmenting activity of a coumarin-derivative known as daphnetin. After chemical characterization by LC-UV and MS analysis, the *Daphne odora* extract was in vitro tested focusing on its inhibitory effect on the melanin biosynthesis pathway suggesting a potential use of this molecule as a depigmenting agent.

The interesting researches of this Special Issue reveal as the study of plant metabolome, performed with “omics” platform together with traditional technologies could provide deep insight into plant cellular pathway. The identification and the characterization molecular components with specific activity could be usefully employed in plant functional genomics with important application for both quality and yield crop increase and for medical purpose.
